# Variable Gene Expression in Human Ganglia Latently Infected with Varicella-Zoster Virus

**DOI:** 10.3390/v14061250

**Published:** 2022-06-09

**Authors:** Peter G. E. Kennedy, Paul Montague

**Affiliations:** 1 Institute of Neuroscience and Psychology, University of Glasgow, Glasgow G61 1QH, UK; 2 Institute of Infection, Immunity and Inflammation, College of Medical, Veterinary and Life Sciences, University of Glasgow, Glasgow G61 1QH, UK; paul.montague@glasgow.ac.uk

**Keywords:** varicella-zoster, virus, latency, gene expression, ganglion, autopsy

## Abstract

Varicella-Zoster virus (VZV) is a pathogenic human herpes virus that causes varicella (“chicken pox”) as a primary infection, following which it becomes latent in neuronal cells in human peripheral ganglia. It may then reactivate to cause herpes zoster (“shingles”). Defining the pattern of VZV gene expression during latency is an important issue, and four highly expressed VZV genes were first identified by Randall Cohrs in 1996 using cDNA libraries. Further studies from both his and other laboratories, including our own, have suggested that viral gene expression may be more widespread than previously thought, but a confounding factor has always been the possibility of viral reactivation after death in tissues obtained even at 24 h post-mortem. Recent important studies, which Randall Cohrs contributed to, have clarified this issue by studying human trigeminal ganglia at 6 h after death using RNA-Seq methodology when a novel spliced latency-associated VZV transcript (VLT) was found to be mapped antisense to the viral transactivator gene 61. Viral gene expression could be induced by a VLT-ORF 63 fusion transcript when VZV reactivated from latency. Prior detection by several groups of ORF63 in post-mortem-acquired TG is very likely to reflect detection of the VLT-ORF63 fusion and not canonical ORF63. The contributions to the VZV latency field by Randall Cohrs have been numerous and highly significant.

## 1. Introduction

The issue of which viral genes are expressed during Varicella-Zoster virus (VZV) latency in human ganglia has been addressed by several groups over the last few decades. VZV is a ubiquitous pathogenic human herpes virus that causes varicella (“chicken pox”) as a primary infection. Then, after a highly variable latent period in neurons in human trigeminal ganglia (TG), dorsal root ganglia (DRG) and peripheral autonomic ganglia, the virus may reactivate, either spontaneously or following various triggering factors to produce herpes zoster (“shingles”), which is a very painful vesicular rash occurring in a dermatomal distribution [1,2]. Herpes zoster may sometimes be followed by post-herpetic neuralgia (PHN), which causes severe pain in the affected dermatome that may prove highly resistant to all therapy [2]. It has been established that during the process of VZV latency the virus is located predominantly in neurons in the affected ganglia [3].

Professor Randall Cohrs of Colorado University made significant contributions to the investigation and our understanding of VZV transcription during human ganglionic latency. This question is important as knowledge of the extent of VZV gene transcription is essential for a better understanding of the mechanism of VZV latency—especially as the biological functions of some of the VZV genes are known [4]. In an early study, Cohrs and his colleagues [5] constructed a cDNA phage library from poly(A)+ RNA obtained from latently infected human ganglia obtained at autopsy within 24 h of death and detected VZV-specific inserts by PCR. Using this then-innovative technique they detected VZV gene 21-specific sequences in the ganglionic samples. This was followed up two years later [6] by the detection of VZV genes 21, 29, 62 and 63 from a human ganglia cDNA library, consistent with the previously reported detection of VZV genes 29 and 62 by another group using Northern blot analyses [7]. This was the first report of VZV gene 63 transcription during latency, and expression of this viral transcript has become a key hallmark of VZV latency. These four VZV latency-associated transcripts were also found to be present in post-mortem human TG in an extensive subsequent study in [8] using PCR in situ hybridisation technology, when it was also found that VZV gene 4 was also detectable in a few subjects. Three years later, in a collaboration between the laboratories of Dr Cohrs and the author, it was reported that both transcription and translation of VZV gene 66 could be demonstrated in latently infected human TG [9]. In view of this novel finding, it was suggested that the prevention of immediate-early (IE) 62 import to the nucleus by VZV 66-pk phosphorylation is one possible mechanism by which VZV latency is maintained.

One of the key problems with these types of study is that it is possible that viral reactivation may occur as a result of the death of the individual itself. This is an inevitable concern with any ganglionic specimen obtained at post-mortem around 24 h after death, which is the case with most of these studies. For this reason, it has long been suspected by some experts in the field that the extent of viral transcription detected in such studies may reflect possible viral reactivation as well as true viral latency. A clear indication of this possibility was provided by a subsequent study from Dr Cohr’s laboratory. This study used a multiplex reverse transcription (RT)-PCR assay that allowed the rapid and sensitive detection of transcripts corresponding to all 68 unique varicella-zoster virus (VZV) open reading frames (ORFs) in five amplification reactions [10]. It was found that, while the detection of VZV gene 63 transcripts was the most frequently detected, a total of 10 viral transcripts were detected—including ORFs 4, 11, 29, 40, 41, 43, 57, 62, 63 and 68. This represented the expression of IE, early and late VZV transcripts, which was highly suggestive of some degree of VZV reactivation as well as genuine latency. The recent report of Depledge and colleagues [11] obtained post-mortem ganglia within about 6 h after the death of the individuals—a much earlier time than before, thereby avoiding the potential problem of possible viral reactivation having taken place. They detected only two viral transcripts using a highly sensitive enriched RNA-Seq method. As well as detecting VZV ORF 63, which was expected, these workers also detected a spliced latency-associated VZV transcript (VLT) that mapped antisense to the viral transactivator gene 61 [11]. These findings are considered further below. It is significant that Dr Cohrs was an author on this manuscript, reflecting his underlying desire to obtain the most accurate picture possible of a biological process.

In order to obtain further evidence of VZV reactivation, as well as latency, in post-mortem TG tissues obtained at 24 h after death, we carried out a comprehensive and systematic study of all 68 transcripts using a nested PCR stratagem.

## 2. Materials and Methods

### 2.1. Tissues and RNA Extraction

**Figure 1:**
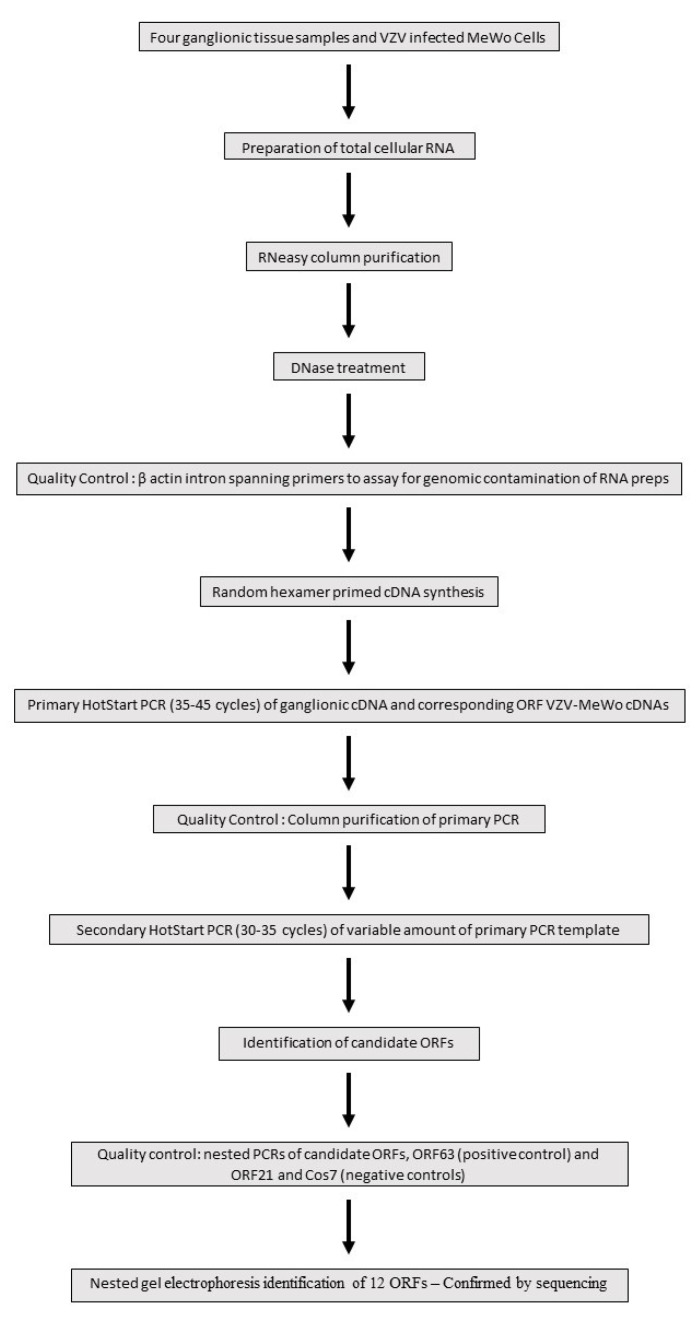
Flow chart depicting key stages in the nested PCR analysis to identify VZV genes expressed in ganglionic tissue.

Four human ganglionic tissues (trigeminal ganglia—TG) were kindly donated and arbitrarily designated S24, S27, S35 and S36 by the Medical Research Council HIV Brain and Tissue Bank in Edinburgh, Scotland, with appropriate ethical clearance. All tissues were obtained at autopsy at around 24 h after death (and not earlier than this). To our knowledge, all four subjects—who were totally anonymised—were immunocompetent and had no recent history of herpes zoster. As depicted in Figure 1, the total cellular RNA was extracted from the four human trigeminal ganglia samples using the commercial reagent RNABee (ams Biotechnology), purified through a silica-membrane RNeasy spin column (Qiagen) and DNase-treated (Ambion). As a quality control check for the presence of genomic contamination in the RNA preps, standard end-point PCRs were performed on the four ganglionic cDNAs using β-actin intron spanning primers which yield different-sized genomic and cDNA PCR products (Figure 1).

### 2.2. Nested RT-PCR Assays

Nested PCR is an established and powerful technique for detecting very low abundance transcripts. A comprehensive and systematic Hot start-based nested PCR screen of the 68 ORFs catalogued at the inception of the study was undertaken. A Hot start approach minimizes non-specific amplification and primer dimer formation by suppressing enzymatic Taq activity until the first denaturation step has been reached. Due to the combined high cycle number employed, (65–80), the nested PCR process is also highly susceptible to template contamination sourced from a VZV laboratory environment. To minimize this potential contamination, nested PCRs were set up in a Class II tissue culture hood using dedicated molecular biology enzymes/chemicals and UV-irradiated plasticware and pipettes. Figure 1 highlights the key stages in a flow chart format of the nested PCR stratagem adopted to screen for VZV ORF genes expressed in ganglionic tissue. Random hexamer primed cDNAs were synthesized using the SuperScript III reverse transcription kit (ThermoFisher Scientific, Waltham, MA, USA). Hot start primary PCRs (JumpStart RedTaq Reaction Mix Sigma) with the outer primers were routinely performed on 5 ng ganglionic cDNAs. cDNA prepared from VZV-infected MeWo cells provided a positive control for the amplification of correctly sized primary PCR products for each ORF (Figure 1). Following electrophoretic analysis of the primary PCR runs, only those ORF samples with a clean background indicative of the absence of non-specific priming were selected to assay for secondary amplification. Using the inner primer pairing, a Hot start (35–45) cycle was performed on an aliquot of the primary PCR product (JumpStart RedTaq Reaction Mix Sigma) and purified using a MSB SpinPCRapace column (Invitek). The synthesis of a correctly sized single secondary PCR product is determined in part by the combination of the primary PCR template load and cycle number. Accordingly, secondary Hot start PCRs comprised of a reduced cycle number (30–35) were tested on a variable amount of purified primary PCR product ranging from 2.5 to 25% (Figure 1). The predicted size differences between the primary and the smaller-sized secondary PCR products were confirmed by 2–3% agarose gel electrophoresis. For these workflow reasons, nested PCRs were initially performed on small batches of up to four ORFs. Positive-scoring secondary PCRs were repeated (*n* ≥ 5). As a final quality control check for each latency candidate, a contemporaneous nested PCR was set up comprised of the candidate ORF, the latency hallmark gene ORF6 as a positive control, ORF21 encoding a nucleocapsid protein and Cos7 cDNA as a representative of a non-human non-neuronal cell type were included as negative controls for VZV gene expression (Figure 1). 

## 3. Results

**Figure 2:**
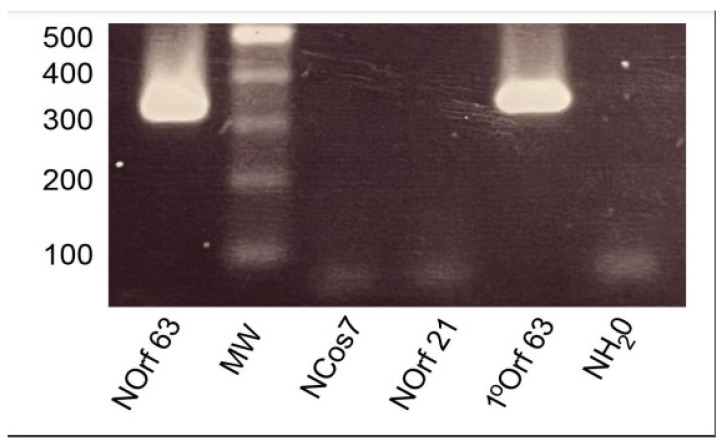
Gel analysis of nested PCR. Nested PCR of ORF 63. Primary PCR amplifies a 386 bp product which yields a 326 bp fragment following a secondary PCR nested run. Nested ORF21, nested Cos7 and nested water controls were negative.

The outcome of this systemic nested PCR of 68 VZV ORFs screen revealed that 12 transcripts were detected in ganglionic tissue as evidenced by gel electrophoresis identification of a correctly sized single secondary PCR product and confirmed by sequencing (Eurofins UK). These are listed in Table 1, correlating ORF activity in each of the four ganglionic samples. Proof of concept of the nested PCR paradigm is illustrated in Figure 2 for ORF63, depicting the 386 bp primary product generated from VZV-infected MeWo cell cDNA and the smaller nested 326 bp PCR fragment. Nested ORF21, Cos7 and water controls were negative.

This panel of 12 active genes was comprised of ORFs 4, 29, 62 and 63—all members of the initial grouping of “classic” latency genes [6,8]—and ORFs 11, 41, and 57, described in the Nagel study [10]. In addition, we report on the activity of a novel cohort comprised of ORFs 32, 37, 42, 58 and 60. However, the activity profile (Table 1) described here raises two main further issues. The first concerns the variation in ORF activity between the four ganglion samples, in that samples S24 and S27 had much less “genetic latency activity” than S35 and S36, while only ORF63—the hallmark latency gene—was active in all samples. The second issue concerns the failure to replicate key published findings. Most notably, we could not detect the classic ORFs 21, 40 and 66 in addition to the ORFs 43 and 68 identified by Nagel and colleagues [10].

## 4. Discussion

Since the pioneering work of Randall Cohrs in 1996 on latent VZV gene expression using cDNA libraries, our knowledge of this process has progressed considerably until the present time. It is noteworthy that Dr Cohrs was a co-author on several of these new analyses over the last 25 years—a testament to his commitment to defining the most accurate analyses of this process and not merely adhering strictly to fixed scientific dogma. It has become clear that latent VZV gene expression is even more restricted than had been previously thought.

In this overview of the subject, we also report some of the findings in our own laboratory—results which clearly demonstrate the widespread VZV gene expression that is detected when human ganglion samples are obtained many hours after death. An interpretation of such findings could be that both true viral latency and also viral reactivation as a consequence of the death of the individual had occurred—a view which is consistent with the study of Nagel et al. [10], where 10 VZV transcripts were detected using a multiplex RT-PCR method, though in neither this nor our own studies were primers targeting the VLT used. The detection of VZV transcripts corresponding to immediate early, early and late VZV genes is possibly suggestive of a degree of viral reactivation. However, it is not all certain that the virus is truly reactivating at these late stages, i.e., regaining the ability to replicate its genome and/or spread to new cells, since there is no definite evidence for this. An obvious difficulty is in relating the predicted function of the novel transcripts that we detected with the latent VZV condition. ORFs 37 and 60 are late genes encoding Glycoproteins H and L, respectively [12], while ORF 42 may be involved in the encapsidation process [13]. The candidacy of the other two transcripts is less of an issue in this regard, as ORF 32 encodes a small polypeptide with unknown function [14], and ORF 58 encodes a nuclear phosphoprotein [15]. At this juncture, it is uncertain whether these ORFs remain “latency candidates” that justify further experimentation or if they can only be classified as transcripts detected in latent tissue and are more likely to be representative of low-level VZV reactivation. Overall, the evidence suggests that the latter possibility is true.

Recent evidence has been based on analyses of human ganglia obtained as early as 6 h after death, which should preclude the possibility of viral reactivation, combined with the application of advanced gene sequencing methodology. Thus, Depledge et al. [11] reported their detection of only two VZV transcripts when they used a very sensitive and enriched RNA-Seq technique on autopsied TG obtained much earlier after death (~6 h) than had been the case in previous studies. As expected, they detected ORF 63 in these ganglia, and detection of this transcript has long been considered to be a hallmark of VZV latency [16]. However, they also detected a novel spliced latency-associated VZV transcript (VLT) that was found to be mapped antisense to the viral transactivator gene 61 [11]. No other VZV transcripts were detected in these tissues. This group also found that VLT is expressed in human TG neurons and encodes a protein which could be detected in both productively infected cells and herpes zoster skin lesions [11]. Moreover, their subsequent studies revealed that viral gene expression could be induced by a VLT-ORF 63 fusion transcript when VZV reactivated from latency [17]. The VLT-ORF63 transcripts appear to be key regulators of the transition of the virus from latency to reactivation. This important study provides the first meaningful investigation of why VLT expression is consistently detected and ORF63 expression intermittently detected in latently infected human ganglia harvested with short post-mortem intervals. The authors concluded that the prior detection by several groups of ORF63 in post-mortem-acquired TG reflected the detection of the VLT-ORF63 fusion rather than canonical ORF63 [17]. It is relevant that VZV small noncoding RNAs antisense to the VLT enhance replication, possibly by regulating its expression [18].

It makes sense at present to focus on the functionality of the VLT, which is clearly a very important novel latency transcript. The fact that the VLT and the VLT-ORF 63 fusion transcripts were the only ones detected so soon after death adds greatly to their significance. Regarding the data presented here, we think it likely that if TG are analysed around 24 h after death, then it is inevitable that a degree of viral reactivation will have already occurred, and this presumably explains why so many VZV transcripts representing all three classes of VZV genes are so frequently detected at these later post-mortem times.

## Figures and Tables

**Table 1:** Activity profile of VZV transcripts detected in the current study.

		Donor Designated Ganglionic Samples			
Classic ORFs	Proposed Function	S24	S27	S35	S36
ORF4	IE Transcriptional activator	–	+	+	+
ORF21	E Replication in culture	–	–	–	–
ORF29	E Transcriptional modulator	+	–	+	+
ORF40	L Encapsidation	–	–	–	–
ORF62	IE Transcriptional activator	+	–	+	–
ORF63	IE Transcriptional activator	+	+	+	+
ORF66	E Protein kinase—Unknown	–	–	–	–
**Nagel et al. [10]**					
ORF11	Tegument protein	–	–	+	+
ORF41	Essential for growth in culture—Unknown	–	+	+	+
ORF43	Essential for growth in culture—Unknown	–	–	–	–
ORF57	Unknown	–	+	–	+
ORF68	Glycoprotein	–	–	–	–
**Novel candidates**					
ORF32	Substrate for ORF47	+	–	+	+
ORF37	L Glycoprotein	–	–	+	+
ORF42	Unknown	+	–	+	–
ORF58	Dispensable for replication—Unknown	–	–	+	–
ORF60	L Protein kinase—viral replication	–	–	+	+

## Data Availability

Not applicable.
